# Distinct phosphorylation requirements regulate cortactin activation by Tir_EPEC _and its binding to N-WASP

**DOI:** 10.1186/1478-811X-7-11

**Published:** 2009-05-06

**Authors:** Elvira Nieto-Pelegrin, Narcisa Martinez-Quiles

**Affiliations:** 1 Microbiología II, Facultad de Farmacia, Universidad Complutense de Madrid, Madrid, Spain

## Abstract

**Background:**

Cortactin activates the actin-related 2/3 (Arp2/3) complex promoting actin polymerization to remodel cell architecture in multiple processes (e.g. cell migration, membrane trafficking, invadopodia formation etc.). Moreover, it was called the Achilles' heel of the actin cytoskeleton because many pathogens hijack signals that converge on this oncogenic scaffolding protein. Cortactin is able to modulate N-WASP activation *in vitro *in a phosphorylation-dependent fashion. Thus Erk-phosphorylated cortactin is efficient in activating N-WASP through its SH3 domain, while Src-phosphorylated cortactin is not. This could represent a switch on/off mechanism controlling the coordinated action of both nucleator promoting factors (NPFs). Pedestal formation by enteropathogenic *Escherichia coli *(EPEC) requires N-WASP activation. N-WASP is recruited by the cell adapter Nck which binds a major tyrosine-phosphorylated site of a bacterial injected effector, Tir (translocated intimin receptor). Tir-Nck-N-WASP axis defines the current major pathway to actin polymerization on pedestals. In addition, it was recently reported that EPEC induces tyrosine phosphorylation of cortactin.

**Results:**

Here we demonstrate that cortactin phosphorylation is absent on N-WASP deficient cells, but is recovered by re-expression of N-WASP. We used purified recombinant cortactin and Tir proteins to demonstrate a direct interaction of both that promoted Arp2/3 complex-mediated actin polymerization *in vitro*, independently of cortactin phosphorylation.

**Conclusion:**

We propose that cortactin binds Tir through its N-terminal part in a tyrosine and serine phosphorylation independent manner while SH3 domain binding and activation of N-WASP is regulated by tyrosine and serine mediated phosphorylation of cortactin. Therefore cortactin could act on Tir-Nck-N-WASP pathway and control a possible cycling activity of N-WASP underlying pedestal formation.

## Background

Enteropathogenic *Escherichia coli *(EPEC) are an important cause of infantile diarrhea, especially in developing countries. EPEC adhere, and cause the local effacement of the microvilli of intestinal epithelial cells, giving raise to so-called attaching and effacing (A/E) lesions. *In vitro*, EPEC attach to infected cells by forming pedestal-like structures enriched in polymerized actin and other host cell proteins [1]. The type III secretion system delivers into host cells the translocated intimin receptor (Tir), which is inserted into the cell plasma membrane such that a loop is exposed on the cell surface that binds to another bacterial protein, the adhesin intimin [2]. This binding induces the clustering of Tir, followed by its phosphorylation on tyrosine residue 474 in the cytoplasmic C-terminal domain. The phosphotyrosine moiety recruits the host cell adaptor protein Nck [3], which binds and presumably activates N-WASP, leading to actin polymerization mediated by the Arp2/3 complex [4]. Although this pathway is recognized as the principal one operating in EPEC, another Nck-independent pathway has also been described in these bacteria [5]. Furthermore, the complexity of EPEC signal transduction is not fully understood [6].

Tir is inserted in the cell membrane, where it adopts a hairpin-loop structure, with both N and C termini projecting into the host cytoplasm [2]. Pedestals are dynamic structures that undergo constant remodeling by cycles of actin polymerization/depolymerization [7]. It is important to understand the contribution of other signals to pedestal formation, not only for EPEC but also for other actin-based processes. For instance, it has been postulated that Tir-Nck signaling mimics the nephrin-Nck-actin pathway [8].

Cortactin is a key regulator of the actin cytoskeleton which plays a crucial role in cell invasion [9] and actin-based motility during the infection of many microbial pathogens [10]. Cortactin possesses an N-terminal acidic domain (NTA) which harbors a DDW motif that activates, albeit weakly, the Arp2/3 complex at branching points [11,12]. The NTA domain is followed by a series of repeat domains that bind filamentous actin (F-actin). The C-terminal SH3 domain of cortactin [13] binds various proteins, such as N-WASP [14], which is a ubiquitously expressed member of the WASP (Wiskott-Aldrich Syndrome) family of proteins. Cortactin can be phosphorylated by tyrosine kinases (Src, Fer, Syk and Abl) and serine/threonine kinases (Erk and Pak) [15]. Src kinase targets tyrosine residues 421, 466 and 482 while Erk phosphorylates serines 405 and 418 [16] which lie in a proline-rich area. Interestingly, a Src family member (Fyn) [17] and Abl kinases phosphorylate Tir [18].

The Arp2/3 complex can be independently activated to initiate actin polymerization by the VCA (Verprolin Cofilin Acidic) domain of WASP members and by both the NTA and F-actin-binding repeats of cortactin. Theoretically N-WASP, cortactin and the Arp2/3 complex can form ternary complexes [19]. Cortactin has been shown *in vitro *to bind and activate N-WASP via an SH3 proline-rich domain interaction [14]. This activation is regulated positively and negatively when cortactin is phosphorylated by Erk and Src respectively. Erk phosphorylation of cortactin or the double mutation S405,418D in cortactin that mimics this phosphorylation enhance the protein's binding to and activation of N-WASP. Conversely, Src phosphorylation inhibits the ability of both Erk-phosphorylated cortactin, and that doubly mutated S405,418D cortactin, to activate N-WASP. Furthermore, phospho-mimetic mutation of the three tyrosine residues targeted by Src (Y421, Y466, and Y482) inhibited the ability of S405,418D cortactin to activate N-WASP. These results led us to hypothesize that Erk phosphorylation liberates the SH3 domain of cortactin from intramolecular interactions, allowing it to synergize with N-WASP in activating the Arp2/3 complex, and that Src phosphorylation terminates cortactin activation of N-WASP. This proposed on/off switching mechanism suggests that phosphorylation of cortactin regulates the accessibility and/or affinity of its SH3 domain towards its targets. 'S/Y model' may be relevant for actin dynamics in multiple cell processes [15] and it may partially explain the coordinated action of cortactin and N-WASP proteins, therefore connecting the two major families of Arp2/3 complex activators. Consistent with this model, recent structural data showed that cortactin adopts a 'closed' globular conformation in which its SH3 domain interacts with the actin-binding repeats [20].

This model has opened up new directions for studies in many cell systems. For example, serine phosphorylation of cortactin has been proposed to be relevant for actin polymerization, while tyrosine phosphorylation have been shown to selectively control adhesion turnover [21]. This suggests that different phosphocortactin forms participate in distinct signaling pathways.

Although it is clear that cortactin participates in pedestal actin dynamics, the underlying mechanism is not well understood. Previous studies have shown that cortactin translocates to EPEC pedestals. Over-expression of truncated forms of cortactin blocks pedestal formation [22]. A follow-up study to this work focused on the role of cortactin domains and Erk/Src phosphorylation, and it confirmed that truncated forms of cortactin exert a dominant negative effect in pedestal formation by EPEC and EHEC (Enterohemorrhagic Escherichia coli). This study suggests that cortactin is recruited through its α-helical region, and the authors conclude that tyrosine phosphorylation is relevant to pedestal formation, whereas serine phosphorylation seems to have no effect on actin assembly underneath the bacteria [23]. However, this conclusion is based exclusively on experiments with phosphorylation-mimicking mutants, without any comparison with the corresponding non-phosphorylatable counterparts.

Nck is not involved in N-WASP recruitment by EHEC. Instead, the EspF_u_/Tccp effector activates N-WASP, thereby mimicking Cdc42 signaling [24,25]. Cantarelli *et al*. have proposed cortactin as the 'missing link' connecting Tir_EHEC _and EspF_u_/Tccp [26]. They showed that EHEC initially induces tyrosine phosphorylation of cortactin and then induces its dephosphorylation, similarly to the transient cortactin phosphorylation during *Helicobacter pylori *infection [27]. However, using the two-hybrid system, they reported that tyrosine-phosphorylated cortactin binds both Tir_EHEC _and EspF_u_/Tccp, and consistent with previously described binding assays using recombinant purified proteins [14], only Erk-phosphorylated cortactin binds N-WASP. Recent *in vitro *studies using cells deficient in N-WASP suggest that cortactin recruitment to EHEC pedestals occurs downstream of EspF_u_/Tccp and N-WASP [28]. It is therefore necessary to gain further insights into cortactin function in both systems. Major unresolved questions include whether cortactin and Tir_EPEC _interact directly, whether cortactin participates in the Tir-Nck-N-WASP pathway, and how cortactin binding partners modulate its nucleating activity on pedestals. Thus, deepening our understanding of the involvement of cortactin on pedestals dynamics is relevant for many reasons.

## Results

### Role of cortactin motifs in pedestal formation

Reduction of cortactin expression by siRNA or over-expression of its isolated SH3 domain, polyproline region or its α-helical region resulted in a drastic decrease in actin-pedestal formation during infection with EPEC [23]. However the role of cortactin's Arp2/3 binding and activating region has not been addressed [23]. Therefore, we investigated its contribution to actin assembly on pedestals using EPEC to infect HeLa cells transiently transfected with GFP-cortactin. Pedestals were visualized by immunofluorescent staining of actin using fluorescent phalloidin and bacteria with DAPI. As previously reported [23], no differences on the number of attached bacteria were observed for the transfectants used (data not shown).

**Figure 1:**
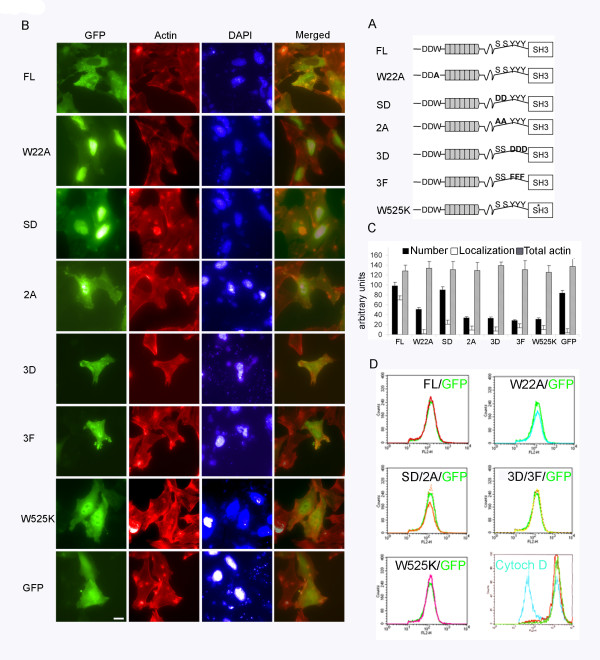
**Effect of the overexpression of WT and cortactin mutants on pedestal formation and total actin content**. (A) Schematic of cortactin domains and mutants under investigation. (B) Cortactin mutants block pedestal formation. Immunofluorescence images of HeLa cells transfected with WT (FL) and cortactin mutants and infected with EPEC for three hours. Mutants used are: Arp2/3 activation mutant (W22A), Erk-phosphorylation-mimicking mutant S405,418D (SD), Erk non-phosphorylatable mutant S405,418A (2A), Src-phosphorylation-mimicking mutant Y421,466,482D (3D), Src non-phosphorylatable mutant Y421,466,482F (3F) and SH3 domain mutant (W525K). GFP staining is shown in green, F-actin is in red, and bacteria and nuclei are in blue. The last column shows merged images of GFP and actin staining. Pictures are at 600× magnification. Scale bar 10 μm. (C) Quantification of pedestal number, cortactin localization and total F-actin content of transfectants. Black bars represent percentages normalized to WT of pedestal formed after 3 hours of infection of HeLa cells expressing GFP-FL and cortactin mutants. White bars represent normalized percentages of localization to pedestals. Grey bars represent the mean fluorescence of total actin content determined by flow cytometry. Experiments were performed at least three times with similar results. (D) Total actin content of HeLa cells transfected with WT and cortactin mutants. Histogram charts of HeLa cells expressing WT and cortactin mutants. Cells were permeabilized and stained with TRITC-phalloidin and total F-actin content was analyzed by flow cytometry. Overlaid histograms show the actin content of cortactin/mutants with respect to GFP transfected control cells (green curve). Pretreatment with cytochalasin D (blue curve) of cells expressing WT cortactin is shown as a control.

The cortactin NTA domain carries a 20DDW22 motif that binds and activates the Arp2/3 complex. Mutation of this motif to 20DDA22, hereafter referred to as W22A, abolished this activity [11]. To determine whether this motif is necessary for pedestal formation we transfected HeLa cells with GFP-W22A. We used wild-type (WT) cortactin (GFP-FL) and GFP alone as controls. As shown in Fig. 1, over-expression of GFP-FL cortactin allowed pedestal formation to levels similar to those in cells expressing GFP. Fig. 1C (black bars) shows normalized percentages and standard deviations for GFP-FL. Results of three independent experiments were considered statistically significant (p < 0.01 by Student's t-test). Since the constructs bear a GFP-tag we were able to simultaneously assess the localization of different cortactin forms (Fig. 1A, B and 1C). We observed GFP-FL cortactin to localize in 70% of pedestals, compared to 4% for GFP-transfected cells (open bars). Importantly, the number of pedestals in cells expressing GFP-W22A mutant was significantly lower than in GFP-FL transfected cells (51% *vs *83%). This result indicates that cortactin W22A exerts a dominant negative effect, which may mean that cortactin binding and activation of the Arp2/3 complex is necessary for pedestal formation.

Cortactin has a C-terminal SH3 domain that binds several proteins. Mutation of a critical amino acid (W525K) abolishes its binding to known targets [29] such as N-WASP [14]. We used this mutant to assess the contribution of the cortactin SH3 domain to pedestal formation; we found that its expression inhibits pedestal formation to an even greater extent than the W22A mutant (31% *vs *51%). This indicates that cortactin W525K mutant exerts a dominant-negative effect, corroborating previous results [23]. In previous work, we described that the cortactin SH3 domain is able to activate N-WASP and we proposed a model for the regulation of N-WASP activation by cortactin, in which cortactin is switched on by Erk phosphorylation of serines 405 and 418, while it is switched off by Src phosphorylation of tyrosines 421, 466 and 482 [14]. Next we repeated the pedestal formation assay with cells expressing the cortactin S405,418D double mutant, which mimics Erk phosphorylation and activates N-WASP *in vitro*, as well as its non-phosphorylatable counterpart (S405,418A). The S405,418D mutant allowed pedestal formation to a similar extent as the WT cortactin (90%) and to a greater extent, although not significantly greater, than the GFP negative control (83%). The phosphoserine-mimicking cortactin mutant accumulated in only 21% of pedestals and showed a weak, diffuse pattern of localization in the cytoplasm and pronounced staining in the nucleus. In contrast, the mutant that abolished Erk phosphorylation (S405,418A) impaired pedestal formation (34%) and its own translocation to them (3%). These results suggest that Erk phosphorylation of cortactin contributes to pedestals formation.

Similarly, we wanted to address the role of Src-mediated phosphorylation of cortactin. We therefore used the phosphotyrosine-mimicking mutant (Y421,466,482D) and the phosphotyrosine deficient mutant (Y421,466,482F). In both cases pedestal formation and location of these constructs on them were impaired (33%/7%; 28%/14%, Fig. 1B and 1C). These results indicate that Src-mediated phoshorylation of cortactin seems to inhibit pedestal formation and that a dynamic phosphorylation of these tyrosine residues play a role in the formation of pedestals.

### Total F-actin content of cells transfected with different cortactin mutants

Although no appreciable changes in the cellular architecture were observed, we wanted to exclude the possibility that over-expression of cortactin mutants induces a general alteration of the actin cytoskeleton. We therefore used flow cytometry to assess the total basal F-actin content of the different transfectants. Fig. 1C (grey bars) and 1D show the mean fluorescence of GFP transfectants, which did not significantly differ based on Student's t-test. As a control, transfected cell were pretreated with Cytochalasin D, a drug known to inhibit actin polymerization (Fig. 1D, bottom right panel). In addition, this experiment allowed us to calculate the transfection efficiency, which was estimated as 60–70%, based on the analysis of the expression of GFP constructs (data not shown).

### EPEC induces N-WASP-dependent tyrosine phosphorylation of cortactin

**Figure 2:**
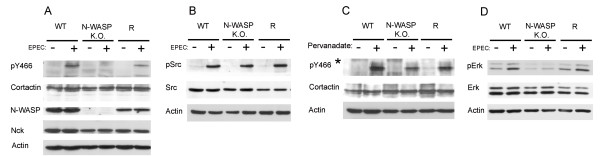
**EPEC-induced tyrosine phosphorylation of cortactin depends on N-WASP**. (A) Tyrosine phosphorylation of cortactin occurs in WT but not in N-WASP-deficient MEFs. WT, N-WASP-deficient and N-WASP reconstituted cells were infected with EPEC for three hours. The level of tyrosine phosphorylation of cortactin was assessed by Western blotting using an antibody against phospho-Y466 cortactin (upper panel). The same membrane was stripped and reprobed with anti-cortactin monoclonal antibody 4F11. N-WASP Western blotting was also performed to confirm the genotype of the MEFs. Actin was used as a loading control. Similar results were obtained in at least three independent experiments. (B) EPEC infection activates Src to similar extends in WT, N-WASP-deficient and R cells. WT, N-WASP-deficient and N-WASP reconstituted cells were infected with EPEC for three hours and analyzed for Src activation (phospho-Y416 Src) (upper panel). Medium and lower panels showed Src and actin blots, respectively. (C) Pervanadate treatment induces a robust phosphorylation of cortactin on tyrosine 466 in WT, N-WASP-deficient and R cells. Lysates of vehicle (DMSO)-treated MEFs and lysates of pervanadate-treated MEFs (diluted 1/500 *) were subjected to SDS-PAGE and Western blotted with antibody against phospho-Y466 cortactin. A second gel was loaded in parallel with the same amount of protein for both types of lysates and blotted for cortactin and actin for protein and loading controls, respectively (medium and lower panels). (D) EPEC infection induces Erk1/2 activation in WT but not N-WASP-deficient MEFs. WT, N-WASP-deficient and R cells were infected with EPEC for three hours. The level of Erk activation was assessed by Western blotting using a mAb specific for activated Erk phosphorylated on Thr202 and Tyr204 (upper panel). Medium and lower panels show Erk and actin blots for Erk protein and loading control respectively. Experiments were performed at least three times.

Contrary to the transient phosphorylation induced by EHEC, EPEC infection of CH7 mouse fibroblasts induces tyrosine phosphorylation of cortactin [26]. Src has been shown to phosphorylate cortactin on tyrosines Y421, 466 and 482 [30], which decreases cortactin affinity for N-WASP *in vitro *[14]. In addition, N-WASP-deficient cells do not form pedestals [31]. These observations prompted us to examine the phosphorylation status of cortactin in WT mouse embryonic fibroblasts (MEFs), MEFs deficient in N-WASP and MEFs deficient in N-WASP in which the protein was later restored through retroviral transduction (rescued, R). First, we performed Western blotting control experiments to assess the expression of N-WASP, cortactin and actin (Fig. 2A).

Fig. 2A shows that EPEC induces phosphorylation of tyrosine 466 at 3 hours of infection in WT MEFs, as detected using an antibody against phospho-Y466-cortactin. This result was corroborated using a second phospho-specific antibody (pY421, data not shown). Unexpectedly, phosphorylation of tyrosine residue 466 was not induced in N-WASP-deficient cells. This result suggests that tyrosine phosphorylation of cortactin during EPEC infection depends on the presence of N-WASP. To verify this, we infected R cells with EPEC and examined levels of phosphoY466-cortactin. Fig. 2A shows that N-WASP re-expression partially restored cortactin tyrosine phosphorylation levels. In three independent experiments the normalized average induction was 1 ± 0.2 for WT cells, 0 for N-WASP-deficient cells and 0.5 ± 0.1 for R cells. This supports the idea that EPEC-induced tyrosine phosphorylation of cortactin in cells requires N-WASP.

Given the absence of cortactin tyrosine phosphorylation in EPEC-infected N-WASP-deficient cells, we then checked Src activation, using a commercially available phospho-active Src antibody (pY416). Fig. 2B demonstrated that equal activation of Src was achieved during EPEC infection in all cell types studied, while, as expected, the levels of total Src remained constant during infection. This result showed that the lack of cortactin phosphorylation in N-WASP-deficient cells was not due to a block in Src activation. As a further control, we treated the cells with pervanadate and observed robust phosphorylation of cortactin tyrosine 466 (Fig. 2C, upper panel).

Similarly we sought to establish the activation status of Erk in EPEC-infected cells. We used a phospho-specific monoclonal antibody that detects the activated form of Erk1/2 (anti pThr202-pTyr204). EPEC induced the activation of Erk on WT MEFs (Fig. 2D first lane), in agreement with a previous report on T84 epithelial cells [32]. However, infection of N-WASP-deficient cells showed reduced activation of Erk which was recovered in R cells. This result implies that Erk is activated by EPEC and may phosphorylate cortactin *in vivo*. More importantly, N-WASP is absolutely required for the induction of Erk activation at 3 hours of infection. However, WT MEFs treated with ERK inhibitors PD98056 or U0126 showed no difference in the number of pedestals formed ([23] and data not shown).

### Tir binds cortactin and induces the latter to nucleate actin *in vitro *through an Arp2/3 complex-mediated pathway

The bacterial protein called Tir initiates what is considered to be the principal signaling cascade, which consists of Tir clustering and concomitant phosphorylation on its tyrosine 474, which then recruits Nck. The latter presumably binds N-WASP to initiate Arp2/3 complex-mediated actin polymerization [1]. We wanted to gain insights into how cortactin functions in pedestal signaling. Our initial hypothesis was that cortactin and Tir interact directly. Therefore we used the Scansite database [33] to search for motifs in the Tir sequence to which cortactin SH3 domain could bind. We found a consensus motif (NNS**I**PPA**P**PL**P**SOTD) centered on proline 20 of Tir.

**Figure 3:**
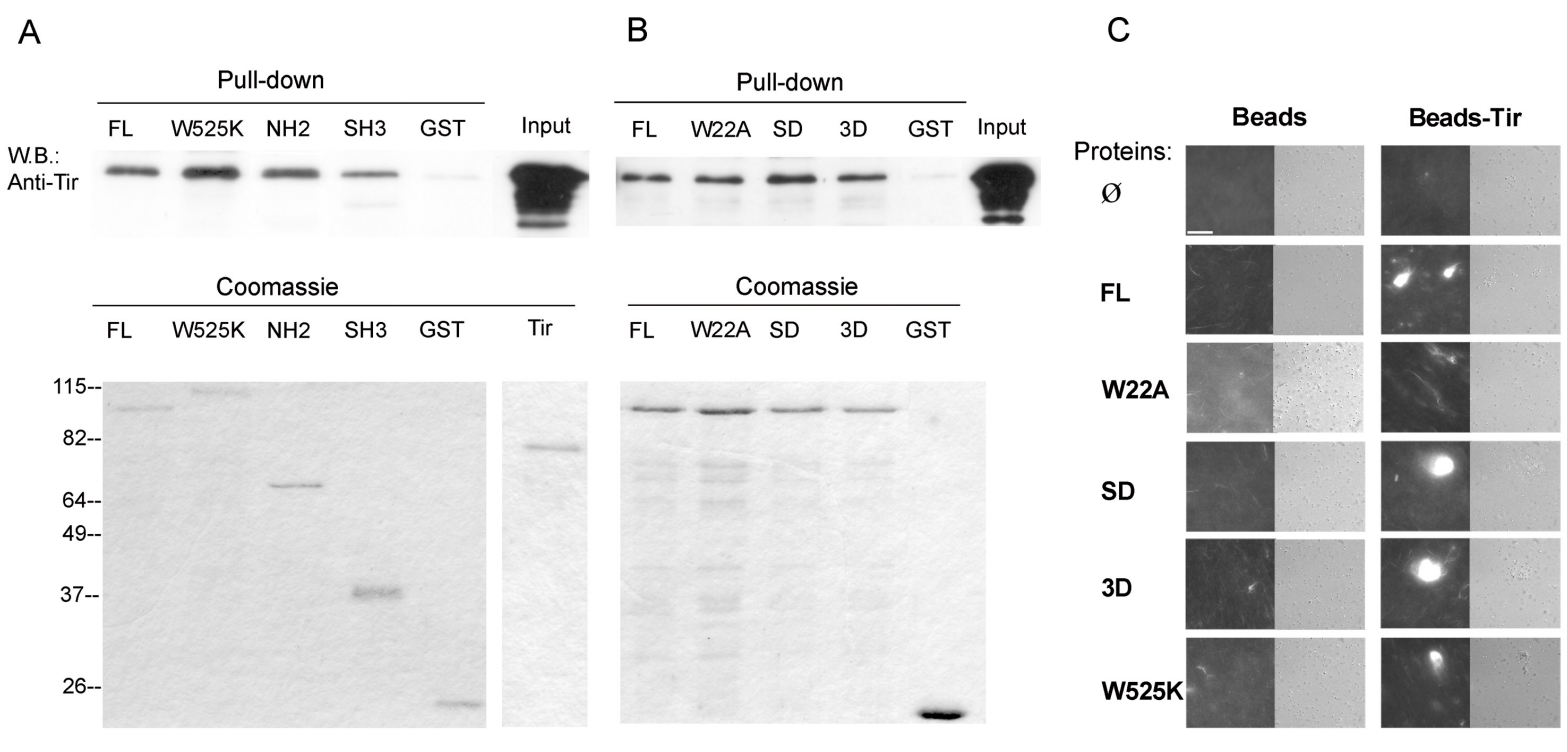
**Tir binds cortactin and promotes its activation of Arp2/3-mediated actin polymerization**. (A) Cortactin binds Tir through the N-terminal and the SH3 domains *in vitro*. Recombinant Tir was incubated with WT cortactin (FL) and with the three mutants expressed as GST fusion proteins: the SH3 domain mutant (W525K), the N-terminal (NH2) domain and the SH3 domain (SH3). GST alone served as a negative control. The pull-downs were subjected to SDS-PAGE and blotted with anti-Tir monoclonal antibody. In the last line, one-fifth of the total amount of Tir that was used as input per pull-down sample (upper left panel). Coomassie staining of proteins is also shown (lower panels). Half of the GST-proteins and on-fifth of Tir that was used in the pull-downs was stained with Coomassie blue. (B) Cortactin binds Tir independently of phosphorylation *in vitro*. Recombinant Tir was incubated with WT cortactin (FL) and with the three mutants expressed as GST fusion proteins: Arp2/3 domain mutant (W22A), Erk-phosphorylation-mimicking mutant (SD) and Src-phosphorylation- mimicking mutant (3D). GST alone served as a negative control (right upper panel). Coomassie staining of proteins is also shown (lower panels). Half of the GST-proteins and one-fifth of Tir that were used in the pull-downs were stained with Coomassie blue (shown in A). (C) Immunofluorescence images of Tir-coupled beads incubated with actin, Arp2/3 and cortactin/mutants. Carboxilate beads (diameter 1 μm) uncoupled (left panels) or coupled to Tir protein (right panels) were incubated with a solution of 500 nM WT cortactin or cortactin mutants proteins for 1 hour. Then, actin and Arp were added and incubated at RT to allow actin polymerization. After 1 hour TRITC-phalloidin was added, and the samples were observed immediately on a microscope. Pictures were taken at 600× magnification. Scale bar 40 μm.

We first performed pull-down experiments with purified recombinant Tir [34] and cortactin proteins (Fig. 3). We produced WT GST-Tir that was purified using GSH beads and treated with PreScission enzyme, which excised Tir and at the same time removed the GST tag (Fig. 3, Coomassie gel). This Tir protein was used as the input in pull-down experiments with GST-cortactin. The first line of Fig. 3A shows that cortactin binds Tir *in vitro*.

To map the domains involved in the interaction, we performed pull-down experiments using cortactin mutants as follows: full-length W525K (mutated SH3 domain), the N-terminus (NH2, residues 1–333), and the isolated SH3 domain (SH3, residues 458–546). GST was used as a negative control. In agreement with our initial hypothesis, the isolated SH3 domain of cortactin bound Tir. However, the N-terminal domain of cortactin also bound Tir (Fig. 3A, third line). This unforeseen interaction was confirmed in experiments with cortactin carrying the point mutation, W525K in the SH3 domain (Fig. 3A). We obtained similar results using as input the Tir phospho-mimicking mutant TirY474D (TirD, data not shown). Next we tested the cortactin S405,418D (SD) and Y421,466,482D (3D) mutants which were similar to the WT form in their ability to bind both Tir (Fig. 3B) and TirD (data not shown). These results demonstrate that cortactin and Tir interact directly *in vitro*, that this interaction involves both the N-terminal part and the SH3 domain, and that it appears to be independent of cortactin phosphorylation.

Given the direct interaction between Tir and cortactin, we wondered whether Tir can activate the ability of cortactin to promote Arp2/3-mediated actin polymerization. We coupled recombinant Tir protein (or TirD, data not shown) to 1 μm beads, and then we washed the beads with Xb buffer and blocked them in Xb buffer containing 1% BSA. Next we incubated them with purified Arp and actin in Xb buffer containing WT and cortactin mutants. Fig. 3C shows that Tir activated WT cortactin and both SD and 3D mutants. Similar results were obtained for TirD (data not shown). The W525K mutant was also activated, although weakly. As expected, W22A cortactin was not activated, indicating that the effect was mediated by cortactin activation of the Arp2/3 complex. As a negative control we used naked beads that showed no activation. Conversely, experiments in which cortactin and its mutants were coupled to GSH beads showed similar results (data not shown). These results indicate that Tir activates the ability of cortactin to promote Arp2/3-mediated actin polymerization *in vitro*.

### Cortactin binding to Tir in N-WASP-deficient cells infected by EPEC

**Figure 4:**
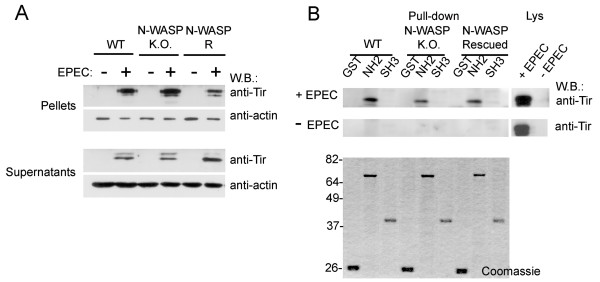
**Cortactin binding to Tir in N-WASP-deficient cells infected by EPEC**. (A) Tir is abundant in membrane-enriched fractions. EPEC-infected and uninfected P100 monolayers of WT, N-WASP-deficient, and R MEFs were lysed in imidazole buffer, fractionated, subjected to SDS-PAGE and blotted with anti-Tir mAb. As previously described, Tir appears as a doublet whose upper band is enriched in the membrane fractions (pellets). The signal from the anti-actin antibody is shown as a loading control. (B) Cortactin binds Tir in cells through its N-terminal domain. Cell lysates of uninfected and EPEC- infected WT, N-WASP-deficient, and R (R) MEFs were analyzed in pull-down experiments using GST fusion proteins as follows: cortactin N-terminal truncation mutant (NH2), the isolated SH3 domain (SH3), and GST alone. Western blotting with anti-Tir monoclonal antibody revealed a unique band corresponding to the slower-moving band. This band was present only in the NH2 pull-down conducted on all three types of lysates. These experiments were performed at least three times.

Because cortactin binds directly both Tir (Fig. 3) and N-WASP [14], we analyze cortactin-Tir interaction in N-WASP-deficient cells. Since those cells do not form pedestals [35], we wondered if Tir would be present at similar levels to WT cells. To address this question, we used a previously described fractionation protocol that enriches in Tir-containing membranes ([36] and Materials and Methods). As shown in Fig. 4A, as expected Tir was enriched in the pellets compared to supernatants, as detected by western-blotting with anti-Tir mAb. We observed that a band with slower electrophoretic motility was the predominant form of Tir in the pellets, which represents fully-modified Tir [37]. WT and N-WASP-deficient cells presented detectable amounts of mature Tir that was slightly reduced on R cells.

FL cortactin has a closed conformation [14,20]. Therefore, we decided to use N-terminal cortactin (NH_2_), the SH3 domain (SH3) and GST as a negative control to perform pull-down experiments with lysates of EPEC infected and uninfected WT, N-WASP-deficient and R cells. Western blotting in Fig. 4B shows that NH_2 _bound Tir in EPEC infected but not uninfected cells, with no appreciable differences between WT, N-WASP and R cells. Similar results were obtained with total cell lysates although longer exposure times where necessary to detect Tir (data not shown).

In contrast, neither the isolated SH3 domain nor the GST negative control bound Tir in any of the cells types used. In view of these results, we can conclude that in cells, cortactin binds Tir primarily through its N-terminal region. To test whether the SH3 domain of cortactin prefers to bind N-WASP over Tir, we performed pull-downs with clarified total lysates, and we then stripped and reprobed the blots with anti-N-WASP antibody. This approach was necessary because the pellets did not contain easily detectable levels of N-WASP (data not shown). As previously described [14], the SH3 domain of cortactin was able to pull-down N-WASP in WT cells but not N-WASP-deficient cells (data not shown). This argues in favor of the conclusion that the N-terminal region of cortactin is involved in binding Tir, while the SH3 domain is involved in binding N-WASP.

## Discussion

Cortactin is a scaffold protein implicated in many cellular processes since it directly contributes to cytoskeleton remodeling. Cortactin also has oncogenic properties due to its role in controlling invadopodia formation and cell migration. Moreover, cortactin has emerged as an important target of numerous pathogens, including enteropathogenic *E. coli *that manipulate the actin cytoskeleton in order to invade the host and propagate there [10]. EPEC cause severe diarrheal disease in humans by colonizing the gut mucosa and producing A/E lesions. EPEC attach to mammalian intestinal cells and induce reorganization of the actin cytoskeleton into 'pedestal-like' structures underneath the bacteria. A crucial event for pedestal formation is the insertion into the host-cell membranes of the EPEC effector Tir, which is initially injected into the cell by a type III secretion system. Tir mimics signaling pathways of the infected cell. Thus it can serve as a powerful model system to study eukaryotic transmembrane signaling [38]. In fact, the Tir-Nck-N-WASP pathway is the principal one through which actin polymerizes in EPEC pedestals. Those reasons prompted us to study cortactin signaling during EPEC infection using N-WASP-deficient cells. Although cortactin localizes to pedestals and its truncated forms exert a dominant negative effect, its function is not clear. For example, does cortactin on its own contribute to actin polymerization in pedestals? Our transfection experiments with the GFP-W22A cortactin point mutant demonstrate that cortactin binding and activation of the Arp2/3 complex is necessary for pedestal formation, which suggests that cortactin indeed contributes to efficient actin polymerization. A complementary study used a similar approach to examine the role of cortactin domains on pedestal formation [23]. It reported identical results to ours regarding WT cortactin and the mutant W525K. However, the W22A mutant was not studied in that work.

To address the role of Erk and Src phosphorylation of cortactin, we used both phosphorylation-mimicking and non-phosphorylatable mutants; previous studies have used only the former [23]. Therefore, we were able to detect a 'neutral' effect on pedestal formation of mutant that mimics phosphorylation by Erk, while the Erk non-phosphorylatable form blocked pedestal formation. Thus, we conclude that phosphorylation of cortactin by Erk may positively regulate pedestal formation. Our conclusion is also supported by other studies: over-expression of a mutant of cortactin mimicking phosphorylation on serine enhanced invadopodia formation in cells in which endogenous cortactin expression had previously been reduced by siRNA [39]. We could not use a similar approach in the present study because the cells detached and died upon EPEC infection (data not shown). The presence of endogenous cortactin may explain why the SD mutant did not lead to significantly more pedestals than WT, although an increase was detectable. Experiments with cortactin-deficient cells may provide the definitive answer to this question. In contrast, phosphoserine-mimicking cortactin accumulated in only one-fourth of pedestals and showed weak diffuse staining in the cytoplasm and a strong nuclear staining. We do not understand this distribution, and we are currently investigating it.

**Figure 5:**
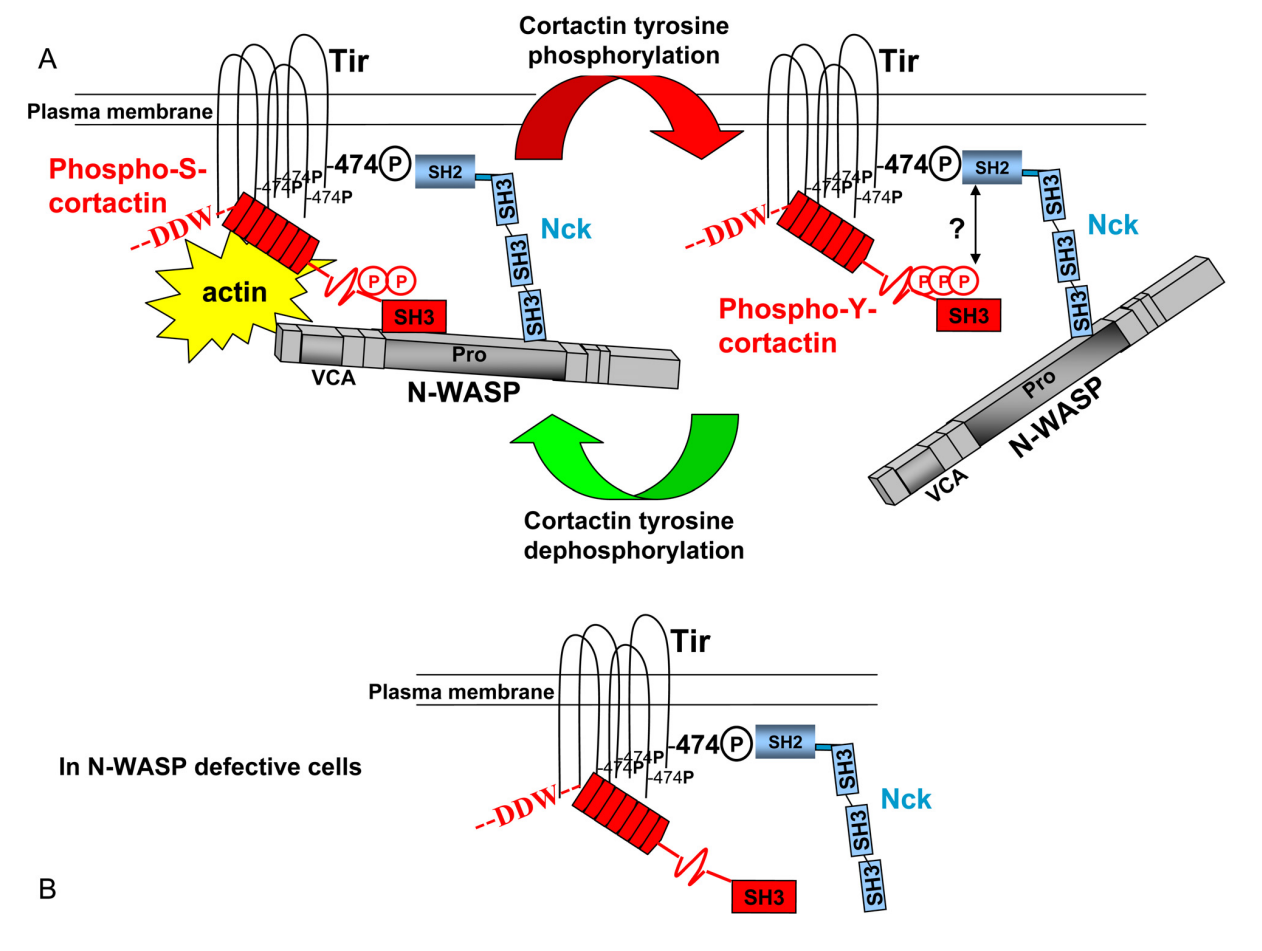
**Model of cortactin action on EPEC pedestals**. (A) Model of coordinated action by cortactin and N-WASP on EPEC pedestals. In theory, cortactin may bind Tir and N-WASP simultaneously, via its N-terminal and SH3 domain interaction respectively. EPEC-induced tyrosine phosphorylation of cortactin would terminate cortactin interaction with N-WASP but not with Tir. This could constitute a cyclical regulatory mechanism of actin polymerization on EPEC pedestals. Phosphotyrosine-cortactin might as well compete for the SH2 domain of Nck, thus uncoupling Tir from the Nck/N-WASP complex. (B) In the absence of N-WASP protein, cortactin would only interact with Tir, which would not be sufficient for pedestal formation.

Src phosphorylates cortactin on positions Y421, 466, and 482 [30]. Therefore we used phosphorylation-mimicking and non-phosphorylatable triple mutants. In both cases pedestal formation was impaired, as well as the accumulation of the mutant proteins to the pedestals that did form. These results indicate that phoshorylation of cortactin by Src inhibits pedestal formation. The same conclusion was reached using the double Y421,466D mutant which partially mimics Src phosphorylation [23], which further supports the idea that cortactin phosphorylated on tyrosine inhibits pedestal formation. The fact that both Src mimicking and non-phosphorylatable cortactin forms inhibited the formation of pedestals might indicate that a dynamic phosphorylation of these tyrosine residues play a role in the formation of pedestals (Fig. 5). Finally, we can exclude that the effects on pedestals were due to changes in the total actin content of the transfectants, because the content was similar for all transfectants examined (Fig. 1). This argues that our results on pedestal formation reflect the specific effects of phosphorylation or lack of phosphorylation.

A crucial finding of this study is that tyrosine phosphorylation of cortactin is abrogated in N-WASP-deficient cells but recovered by N-WASP re-expression (Fig. 2). In agreement with these results, preliminary data using an antibody against cortactin phosphorylated on serine 405 show that EPEC induces serine phosphorylation of cortactin, which is not up-regulated in EPEC infected N-WASP-deficient cells (Narcisa Martínez-Quiles and Steffen Backert, unpublished results). Importantly, the lack of cortactin tyrosine phosphorylation was not due to a defect on Src activation. We think that only the fraction of cortactin that has translocated to the pedestals is available for serine and tyrosine phosphorylation. These findings strongly suggest a coordinated action of cortactin and N-WASP during pedestal formation, consistent with the on/off switching mechanism by which cortactin activates N-WASP *in vitro *[14]. A remaining question is whether cortactin is phosphorylated sequentially, e.g. serine followed by tyrosine phosphorylation. The lack of induction of cortactin phosphorylation in N-WASP-deficient cells should prove to be examined in many signaling transduction studies.

On the other hand, most studies have used inhibitors to establish the role of kinases on pedestal signaling and have mainly focused on Tir phosphorylation [25]. To our knowledge this is the first report that establishes the status of Src activity during pedestal formation on N-WASP-deficient cells. Another conclusion that can be drawn is that Erk and Src kinases become activated in response to different signals. Thus Src is not affected by ablation of N-WASP whereas Erk activity is seriously compromised (Fig. 2). Erk activation is shut off sooner in N-WASP-deficient cells than in WT cells as seen in timing experiments. In contrast, the basal level of cortactin phosphorylated on serine was higher in Nck-deficient cells than in WT cells, and it was increased upon EPEC infection (data not shown). Thus we can be confident that the lack of cortactin phosphorylation is not merely due to the lack of pedestals, since cells deficient in either N-WASP or Nck do not form pedestals [3,31].

We report here that cortactin and Tir bind each other directly *in vitro *(Fig. 3A). Our initial hypothesis was that they would interact directly through the SH3 domain cortactin, because Tir possess a consensus motif centered on proline P20. Indeed, the SH3 domain was able to bind Tir, but unexpectedly, the NH2 domain was also found to bind Tir (Fig. 3). In addition, we did not detect differences in the affinity binding of mutants that mimic phosphorylation by Erk and Src, which contrast our previous binding studies in which a mutant that mimics phosphorylation by Erk was found to bind preferentially to N-WASP [14]. These results demonstrated that cortactin and Tir interact directly *in vitro*, through both the N-terminal region and the SH3 domain of cortactin, and this interaction seems to occur independently of cortactin phosphorylation. In agreement with this conclusion, experiments using a two-hybrid system show that both the N-terminal region and the SH3 domain of cortactin bind Tir_EHEC_. However, a major difference with our results is that only tyrosine-phosphorylated cortactin bind Tir_EHEC_, which contrasts with the transient phosphorylation of cortactin induced by EHEC. Both findings were reconciled by suggesting cortactin and Tir initially bind transiently coincident with the tyrosine phosphorylation of cortactin [26]. In our system, EPEC-infected cells still showed high levels of N-WASP-dependent cortactin phosphorylation three hours after infection. These results highlight the fine-tuned nature of cortactin regulation during EPEC and EHEC infections.

Cortactin can activate the Arp2/3 complex directly through its NTA domain [11,12], and indirectly by using its SH3 domain to activate N-WASP [14]. We wondered whether the binding of Tir to cortactin would activate the latter and promote Arp2/3 complex-dependent actin polymerization. As shown in Fig. 3B, Tir-coated beads activated cortactin. Furthermore, as for the binding, the activation of cortactin by Tir was not affected by the phosphorylation status of cortactin, which further supports the idea that in EPEC signaling, Tir binds and activates cortactin independently of the latter's phosphorylation status. At this point, we favored the conclusion that the relevant contribution underlying cortactin-Tir binding occurs through the N-terminal moiety of cortactin, since our previous studies indicated that phosphorylation of cortactin affects mainly its interaction with partners through the SH3 domain [14]. To test this hypothesis, we used cell lysates that represent a more restrictive scenario with greater similarity to binding conditions *in vivo*. Consistent with our reasoning, the N-terminal region of cortactin bound Tir, whereas the isolated SH3 domain did not in any of the cells type tested. In view of these results, we can conclude that in cells cortactin binds Tir primarily through its N-terminal region, while the contribution of the SH3 domain seems to be irrelevant. Furthermore, the interaction between Tir and cortactin is independent of phosphorylation and does not require N-WASP, since we detected similar levels of interaction in WT, N-WASP-deficient and R cells.

Alternatively, the cortactin SH3 consensus site on Tir may be occupied by other SH3 domains such as tyrosine kinases [40] or the cortactin SH3-domain may have a preference for binding N-WASP. As previously described [14], the SH3 domain of cortactin pulls down N-WASP. This supports the idea that cortactin binds Tir through the N-terminus and N-WASP through the SH3 domain. In this case, phosphorylation should affect only the binding of cortactin to N-WASP; in other words, cortactin phosphorylated on serine would bind both Tir and N-WASP whereas cortactin phosphorylated on tyrosine would bind only Tir.

Both binding and activation experiments were also performed with the Tir phosphorylation mimicking Y474D mutant of Tir (data not shown). The fact that we did not observe significant differences from WT Tir may means: (i) the mutant does not behave like the phosphorylated form or (ii) the binding and activation of cortactin is independent of Tir phosphorylation on residue 474. Further experiments are needed to address this question.

Finally, in our effort to understand what kind of complexes form *in vivo*, we considered all available *in vitro *data concerning the interaction of Tir, Nck, N-WASP and cortactin. Thus Nck binds cortactin only when phosphorylated by Src [41], through an interaction between the phosphotyrosine and the SH2 domain [42]. Therefore since Tir and Nck interact through the single SH2 domain of Nck, formation of a Tir-Nck-cortactin complex appears to be impossible. Cortactin phosphorylated by Src is not able to interact with N-WASP, as shown with recombinant proteins [14] and further corroborated in the two-hybrid assay [26]. That adds to the evidence against the possibility that cortactin bridges both proteins, i.e. Nck-cortactin-N-WASP. This leaves three possible types of complexes: (i) Tir-Nck-N-WASP-cortactin, (ii) Tir-cortactin-N-WASP and (iii) Tir-cortactin. Given the fact that reducing of cortactin expression with siRNA inhibits pedestal formation ([23] and our unpublished results), that EPEC infection induces cortactin phosphorylation in an N-WASP-dependent fashion (Fig. 2), and that Tir binds and activates cortactin (Fig. 3) we conclude that cortactin contributes to the Tir-Nck-N-WASP pathway, possibly by regulating N-WASP activity. In other words, cortactin and N-WASP would act in a complex in this scenario. If we envision pedestals as a dynamic actin structure, and in fact pedestal motility has been shown [7], then it is reasonable to think that proteins promoting actin polymerization would act in a cyclic manner. We speculate that cortactin is a cycling switch for N-WASP in pedestals (Model in Fig. 5).

Deletion of Tir abrogates pedestal formation by EPEC [24] implying that Tir mediates the major but not only pathway for actin assembly in pedestals. Indeed, elegant work has shown that the EPEC effector protein EspF directly activates N-WASP [43]. We can not exclude that cortactin participates in this pathway.

## Conclusion

The function of cortactin in pedestals, and how its function is regulated, seems to differ between EPEC and EHEC. EHEC induces tyrosine dephosphorylation of cortactin [26] whereas EPEC induces its tyrosine phophorylation ([26] and this study). During EHEC infection, the Tir-cortactin interaction was mapped to the N-terminal region of both molecules, but only cortactin phosphorylated by Src bound to Tir_EHEC_.

In our study, cortactin bound directly to Tir_EPEC_ independently of phosphorylation since cortactin mutants mimicking phosphorylation by Erk and Src interacted with Tir, and were activated to a similar extent *in vitro*. This finding further supports our results using EPEC-infected cells that show that the interaction between Tir and cortactin is mediated through the N-terminal part of the cortactin molecule. Our results are compatible with the formation of complexes in which cortactin may interact with Tir via its N-terminal domain and with N-WASP via its SH3 domain. The later interaction would be terminated upon tyrosine phosphorylation of cortactin (Model in Fig. 5).

## Methods

### Cells, bacteria, reagents and antibodies

HeLa human epithelial cells were obtained from ATCC and grown in Iscove's Modified Dulbecco's Media (IMDM) supplemented with 10% FBS (fetal bovine serum). N-WASP-deficient and R mouse embryonic fibroblasts (MEFs) were obtained from Dr. Scott Snapper (Massachusetts General Hospital, Boston, USA) and Nck1/2-deficient MEFs from Dr. Tony Pawson (Samuel Lunenfeld Research Institute, Mount Sinai Hospital, Toronto, Canada). Enteropathogenic *Escherichia coli *(EPEC) E2348/69, as well as monoclonal antibodies against the N- and C-termini Tir (2A5 and 2C3, respectively) were provided by Dr. Brett B. Finlay (University of British Columbia, Vancouver, Canada). Anti-N-WASP antibody was previously described [44]. Commercial antibodies used were: anti-cortactin 4F11 monoclonal antibody, anti-Src GD11 monoclonal and polyclonal anti-phosphoY416 (activated Src) antibodies (Millipore), and anti-actin C4 monoclonal antibody (MP Biomedical). Anti-phospho-cortactin Y466 polyclonal antibody was from Abcam (Cambridge, UK). Polyclonal anti-Erk1/2 (p44/42) and monoclonal anti-phospho-Erk (Thr202/Tyr204, clone E10) antibodies were from Cell Signaling. Anti-rabbit and anti-mouse horseradish peroxidase (HRP) antibodies were from Amersham Pharmacia Biotech.

### Cortactin and Tir constructs

Wild-type cortactin and selected mutants [14] were sub-cloned in frame with GFP at the N-terminus in the plasmid pC2-EGFP (Invitrogen) and verified by sequencing. The constructs used were full-length wild-type cortactin (FL), and the following derivatives: the single point mutants W22A and W525K; the double mutant S405,418D; the triple mutant Y421,466,482D; an N-terminal fragment of cortactin (NH2) containing residues 1–333, and a cortactin fragment (residues 458–546) containing the SH3 domain (SH3) aas. Two new mutants: S405,418A and Y421,466,482F, were generated using PCR and GST-FL as the template with the QuikChange site-directed mutagenesis kit (Stratagene). The Tir Y474D mutant was produced using the QuikChange kit.

### Cell transfection, Western blotting and pedestal formation by EPEC

Cell transfection was carried out using Lipofectamine 2000 (Invitrogen). Briefly, HeLa cells were grown to 60–70% confluence in 6-well plates. Transfections were incubated for 16 hours in medium containing 10% FBS but no antibiotics. Western blotting was done on cells from a single well by directly adding 300 μl of 2× Laemmli buffer and scraping the cells. Samples were homogenized by six passages through a syringe with a 25-gauge needle, followed by centrifugation at 21,000 g for 15 min at 4°C. Samples were resolved by 10% SDS-PAGE and analyzed by Western blotting and developed with ECL (Amersham). Band densitometry was carried out using NIH ImageJ software. Normalization for each experiment was done by first, normalizing actin and next, the protein. The average difference was calculated from three independent experiments and reported as ± standard deviations.

EPEC infections were carried out as follows. Overnight bacterial culture were grown at 37°C with shaking at 200 r.p.m., and 1 μl of culture was added per well of a 6-well-plate. Pedestals were allowed to form for 3 hours in medium containing 10% FBS and no antibiotics at 37°C and 5% CO_2_. When indicated, EPEC was preactivated by incubating a 1/100 dilution of the O/N culture for 2 hours in medium containing 10% FBS and no antibiotics at 37°C and 5% CO_2_; the amount of bacteria from this preactivated culture that was added to wells was 1/8 that of non-preactivated bacteria. Quantification was done by counting the numbers of pedestals of attached bacteria for a total of 100 cells. Experiments were performed at least three times. Statistical analysis was carried out using Student's t-test in Microsoft Excel.

### Immunofluorescence microscopy and determination of total content of F-actin

Cells were fixed with 4% formalin solution (Sigma) at room temperature and permeabilized with 0.1% Triton X-100 for 5 min. After two washes with PBS, cells were blocked with 2% BSA in PBS for 10 min and then sequentially stained with 1 μg/ml tetramethyl rhodamine isotiocianate (TRITC)-phalloidin (Sigma) and DAPI (300 nM). Photographs were taken using a Nikon Eclipse TE 200-U fluorescence microscope equipped with a Hamamatsu camera. Images were processed with Adobe Phothoshop. For determination of total content of F-actin, TRITC-phalloidin was used at 5 μg/ml. As a control, cells were pretreated 15 min with cytochalasin D at 2 μg/ml. Samples were sorted by fluorescence using a FACS Scalibur station. Experiments were performed at least three times. Statistical analysis was carried out using Student's t-test in Microsoft Excel.

### Actin polymerization assays

GST recombinant proteins were produced, purified and, when necessary, treated with PreScission protease according to the manufacturer's recommendations (GE Healthcare) to remove GST. Carboxilate microspheres (1 μm; Polysciences Inc.) were coupled to Tir/TirD in solution (500 nM) and washed once with Xb buffer (10 mM HEPES pH 7.8, 100 mM KCl, 0.1 mM CaCl_2_, 1 mM MgCl_2_, 1 mM ATP) and twice with Xb buffer-1% BSA to block nonspecific interactions. Purified actin (2.5 μM) and Arp (300 nM) were used. Cortactin and its mutants (500 nM) were added to a final volume of 25 μl of Xb buffer. After 1 hour TRITC-phalloidin was added to a final concentration of 3.3 μM. The solution containing the beads was placed on a slide and sealed with paraffin. Pictures were acquired while keeping all relevant parameters fixed (gain, exposure time) to allow for fluorescence intensity comparison. Experiments were performed at least three times.

### Membrane enrichment procedure, pull-down experiments, and pervanadate treatment

After EPEC infection, MEFs were fractionated as described [36] with some modifications. Briefly, MEFs were grown to 70–80% confluence in 150-mm plates and infected with preactivated EPEC. After 3 hours of infection, cells were washed once with ice-cold PBS and rapidly lysed at 4°C by overlaying the cell monolayer for 10 min with 1 ml of buffer containing imidazole (pH 7.4), 250 mM sucrose, protease-phosphatase inhibitor cocktail (Complete™) and phosphatase inhibitor (PhosStop, Amersham). Then the cells were collected using a cell scraper (Sarstedt) and disrupted by six passages through a syringe with a 25-gauge needle, followed by 15 min centrifugation at 3,000 g to remove cellular debris, bacteria and nuclei. Clarified lysates were centrifuged again for 1 hour at 21,000 g to separate the membrane (pellet) from the cytoplasmic fraction (supernatant). Both of these final fractions were stored at -70°C until further use.

For pull-down experiments pellets were resuspended in 400 μl of lysis buffer containing 0.1% Triton-X100, and a fourth was used for each pull-down. GST, the GST-N terminal (NH2) cortactin fragment and the GST-SH3 cortactin domain were produced in BL21 *E. coli*, purified and coupled to GSH-beads. Pull-downs were washed three times with 100 μl of lysis buffer diluted 1:10 in PBS containing 0.05% Tween 20.

Pull-down experiments with recombinant proteins were performed as previously described [14]. When necessary the GST was removed by Precission enzyme treatment (Amersham).

Pervanadate treatment was carried out by mixing 1 mM of NaVO_4 _with 1% H_2_O_2 _and diluting two-fold with IMDM medium for 30 min at 37°C and 5% CO_2_.

## Competing interests

The authors declare that they have no competing interests.

## Authors' contributions

ENP and NMQ performed and designed the experiments. NMQ, the Senior/corresponding author, supervise the experiments and prepare the manuscript with the help of ENP.

## References

[B1] Caron E, Crepin VF, Simpson N, Knutton S, Garmendia J, Frankel G (2006). Subversion of actin dynamics by EPEC and EHEC. Current opinion in microbiology.

[B2] Kenny B, DeVinney R, Stein M, Reinscheid DJ, Frey EA, Finlay BB (1997). Enteropathogenic E. coli (EPEC) transfers its receptor for intimate adherence into mammalian cells. Cell.

[B3] Gruenheid S, DeVinney R, Bladt F, Goosney D, Gelkop S, Gish GD, Pawson T, Finlay BB (2001). Enteropathogenic E. coli Tir binds Nck to initiate actin pedestal formation in host cells. Nature cell biology.

[B4] Kalman D, Weiner OD, Goosney DL, Sedat JW, Finlay BB, Abo A, Bishop JM (1999). Enteropathogenic E. coli acts through WASP and Arp2/3 complex to form actin pedestals. Nature cell biology.

[B5] Campellone KG, Leong JM (2005). Nck-independent actin assembly is mediated by two phosphorylated tyrosines within enteropathogenic Escherichia coli Tir. Molecular microbiology.

[B6] Hardwidge PR, Rodriguez-Escudero I, Goode D, Donohoe S, Eng J, Goodlett DR, Aebersold R, Finlay BB (2004). Proteomic analysis of the intestinal epithelial cell response to enteropathogenic Escherichia coli. The Journal of biological chemistry.

[B7] Sanger JM, Chang R, Ashton F, Kaper JB, Sanger JW (1996). Novel form of actin-based motility transports bacteria on the surfaces of infected cells. Cell Motil Cytoskeleton.

[B8] Blasutig IM, New LA, Thanabalasuriar A, Dayarathna TK, Goudreault M, Quaggin SE, Li SS, Gruenheid S, Jones N, Pawson T (2008). Phosphorylated YDXV motifs and Nck SH2/SH3 adaptors act cooperatively to induce actin reorganization. Mol Cell Biol.

[B9] Weaver AM (2008). Cortactin in tumor invasiveness. Cancer letters.

[B10] Selbach M, Backert S (2005). Cortactin: an Achilles' heel of the actin cytoskeleton targeted by pathogens. Trends in microbiology.

[B11] Uruno T, Liu J, Zhang P, Fan Y, Egile C, Li R, Mueller SC, Zhan X (2001). Activation of Arp2/3 complex-mediated actin polymerization by cortactin. Nature cell biology.

[B12] Weaver AM, Karginov AV, Kinley AW, Weed SA, Li Y, Parsons JT, Cooper JA (2001). Cortactin promotes and stabilizes Arp2/3-induced actin filament network formation. Curr Biol.

[B13] Wu H, Parsons JT (1993). Cortactin, an 80/85-kilodalton pp60src substrate, is a filamentous actin-binding protein enriched in the cell cortex. The Journal of cell biology.

[B14] Martinez-Quiles N, Ho HY, Kirschner MW, Ramesh N, Geha RS (2001). Erk/Src phosphorylation of cortactin acts as a switch on-switch off mechanism that controls its ability to activate N-WASP. Nat Cell Biol.

[B15] Lua BL, Low BC (2005). Cortactin phosphorylation as a switch for actin cytoskeletal network and cell dynamics control. FEBS letters.

[B16] Cosen-Binker LI, Kapus A (2006). Cortactin: the gray eminence of the cytoskeleton. Physiology (Bethesda, Md).

[B17] Phillips N, Hayward RD, Koronakis V (2004). Phosphorylation of the enteropathogenic E. coli receptor by the Src-family kinase c-Fyn triggers actin pedestal formation. Nature cell biology.

[B18] Swimm A, Bommarius B, Li Y, Cheng D, Reeves P, Sherman M, Veach D, Bornmann W, Kalman D (2004). Enteropathogenic Escherichia coli use redundant tyrosine kinases to form actin pedestals. Molecular biology of the cell.

[B19] Weaver AM, Heuser JE, Karginov AV, Lee WL, Parsons JT, Cooper JA (2002). Interaction of cortactin and N-WASp with Arp2/3 complex. Curr Biol.

[B20] Cowieson NP, King G, Cookson D, Ross I, Huber T, Hume DA, Kobe B, Martin JL (2008). Cortactin adopts a globular conformation and bundles actin into sheets. The Journal of biological chemistry.

[B21] Kruchten AE, Krueger EW, Wang Y, McNiven MA (2008). Distinct phospho-forms of cortactin differentially regulate actin polymerization and focal adhesions. Am J Physiol Cell Physiol.

[B22] Cantarelli VV, Takahashi A, Yanagihara I, Akeda Y, Imura K, Kodama T, Kono G, Sato Y, Iida T, Honda T (2002). Cortactin is necessary for F-actin accumulation in pedestal structures induced by enteropathogenic Escherichia coli infection. Infect Immun.

[B23] Cantarelli VV, Kodama T, Nijstad N, Abolghait SK, Iida T, Honda T (2006). Cortactin is essential for F-actin assembly in enteropathogenic Escherichia coli (EPEC)- and enterohaemorrhagic E. coli (EHEC)-induced pedestals and the alpha-helical region is involved in the localization of cortactin to bacterial attachment sites. Cellular microbiology.

[B24] Campellone KG, Rankin S, Pawson T, Kirschner MW, Tipper DJ, Leong JM (2004). Clustering of Nck by a 12-residue Tir phosphopeptide is sufficient to trigger localized actin assembly. The Journal of cell biology.

[B25] Garmendia J, Phillips AD, Carlier MF, Chong Y, Schuller S, Marches O, Dahan S, Oswald E, Shaw RK, Knutton S (2004). TccP is an enterohaemorrhagic Escherichia coli O157:H7 type III effector protein that couples Tir to the actin-cytoskeleton. Cellular microbiology.

[B26] Cantarelli VV, Kodama T, Nijstad N, Abolghait SK, Nada S, Okada M, Iida T, Honda T (2007). Tyrosine phosphorylation controls cortactin binding to two enterohaemorrhagic Escherichia coli effectors: Tir and EspFu/TccP. Cellular microbiology.

[B27] Selbach M, Moese S, Hurwitz R, Hauck CR, Meyer TF, Backert S (2003). The Helicobacter pylori CagA protein induces cortactin dephosphorylation and actin rearrangement by c-Src inactivation. The EMBO journal.

[B28] Mousnier A, Whale AD, Schuller S, Leong JM, Phillips AD, Frankel G (1998). Cortactin recruitment by enterohemorrhagic Escherichia coli O157:H7 during infection in vitro and ex vivo. Mol Cell Biol.

[B29] Du Y, Weed SA, Xiong WC, Marshall TD, Parsons JT (1998). Identification of a novel cortactin SH3 domain-binding protein and its localization to growth cones of cultured neurons. Mol Cell Biol.

[B30] Huang C, Liu J, Haudenschild CC, Zhan X (1998). The role of tyrosine phosphorylation of cortactin in the locomotion of endothelial cells. The Journal of biological chemistry.

[B31] Lommel S, Benesch S, Rottner K, Franz T, Wehland J, Kuhn R (2001). Actin pedestal formation by enteropathogenic Escherichia coli and intracellular motility of Shigella flexneri are abolished in N-WASP-defective cells. EMBO reports.

[B32] Savkovic SD, Ramaswamy A, Koutsouris A, Hecht G (2001). EPEC-activated ERK1/2 participate in inflammatory response but not tight junction barrier disruption. American journal of physiology.

[B33] Yaffe MB, Leparc GG, Lai J, Obata T, Volinia S, Cantley LC (2001). A motif-based profile scanning approach for genome-wide prediction of signaling pathways. Nature biotechnology.

[B34] Race PR, Solovyova AS, Banfield MJ (2007). Conformation of the EPEC Tir protein in solution: investigating the impact of serine phosphorylation at positions 434/463. Biophysical journal.

[B35] Lommel S, Benesch S, Rohde M, Wehland J, Rottner K (2004). Enterohaemorrhagic and enteropathogenic Escherichia coli use different mechanisms for actin pedestal formation that converge on N-WASP. Cellular microbiology.

[B36] Patel A, Cummings N, Batchelor M, Hill PJ, Dubois T, Mellits KH, Frankel G, Connerton I (2006). Host protein interactions with enteropathogenic Escherichia coli (EPEC): 14-3-3tau binds Tir and has a role in EPEC-induced actin polymerization. Cellular microbiology.

[B37] Kenny B, Warawa J (2001). Enteropathogenic Escherichia coli (EPEC) Tir receptor molecule does not undergo full modification when introduced into host cells by EPEC-independent mechanisms. Infect Immun.

[B38] Hayward RD, Leong JM, Koronakis V, Campellone KG (2006). Exploiting pathogenic Escherichia coli to model transmembrane receptor signalling. Nature reviews.

[B39] Ayala I, Baldassarre M, Giacchetti G, Caldieri G, Tete S, Luini A, Buccione R (2008). Multiple regulatory inputs converge on cortactin to control invadopodia biogenesis and extracellular matrix degradation. Journal of cell science.

[B40] Bommarius B, Maxwell D, Swimm A, Leung S, Corbett A, Bornmann W, Kalman D (2007). Enteropathogenic Escherichia coli Tir is an SH2/3 ligand that recruits and activates tyrosine kinases required for pedestal formation. Molecular microbiology.

[B41] Tehrani S, Tomasevic N, Weed S, Sakowicz R, Cooper JA (2007). Src phosphorylation of cortactin enhances actin assembly. Proceedings of the National Academy of Sciences of the United States of America.

[B42] Okamura H, Resh MD (1995). p80/85 cortactin associates with the Src SH2 domain and colocalizes with v-Src in transformed cells. The Journal of biological chemistry.

[B43] Alto NM, Weflen AW, Rardin MJ, Yarar D, Lazar CS, Tonikian R, Koller A, Taylor SS, Boone C, Sidhu SS (2007). The type III effector EspF coordinates membrane trafficking by the spatiotemporal activation of two eukaryotic signaling pathways. The Journal of cell biology.

[B44] Martinez-Quiles N, Rohatgi R, Anton IM, Medina M, Saville SP, Miki H, Yamaguchi H, Takenawa T, Hartwig JH, Geha RS (2001). WIP regulates N-WASP-mediated actin polymerization and filopodium formation. Nature cell biology.

